# Ipsilateral Limb Extension of Referred Trigeminal Facial Pain due to Greater Occipital Nerve Entrapment: A Case Report

**DOI:** 10.1155/2022/9381881

**Published:** 2022-12-02

**Authors:** Byung-Chul Son, Changik Lee

**Affiliations:** ^1^Department of Neurosurgery, Seoul St. Mary's Hospital, College of Medicine, The Catholic University of Korea, Seoul, Republic of Korea; ^2^Catholic Neuroscience Institute, College of Medicine, The Catholic University of Korea, Seoul, Republic of Korea

## Abstract

We report a very rare case of referred pain associated with entrapment of the greater occipital nerve (GON) occurring not only in the ipsilateral hemiface but also in the ipsilateral limb. There is an extensive convergence of cutaneous, tooth pulp, visceral, neck, and muscle afferents onto nociceptive and nonnociceptive neurons in the trigeminal nucleus caudalis (medullary dorsal horn). In addition, nociceptive input from trigeminal, meningeal afferents projects into trigeminal nucleus caudalis and dorsal horn of C1 and C2. Together, they form a functional unit, the trigeminocervical complex (TCC). The nociceptive inflow from suboccipital and high cervical structures is mediated with small-diameter afferent fibers in the upper cervical roots terminating in the dorsal horn of the cervical cord extending from the C2 segment up to the medullary dorsal horn. The major afferent contribution is mediated by the spinal root C2 that is peripherally represented by the greater occipital nerve (GON). Convergence of afferent signals from the trigeminal nerve and the GON onto the TCC is regarded as an anatomical basis of pain referral in craniofacial pain and primary headache syndrome. Ipsilateral limb pain occurs long before the onset of the referred facial pain. The subsequent severe hemifacial pain suggested GON entrapment. The occipital nerve block provided temporary relief from facial and extremity pain. Imaging studies found a benign osteoma in the ipsilateral suboccipital bone, but no direct contact with GON was identified. During GON decompression, severe entrapment of the GON was observed by the tendinous aponeurotic edge of the trapezius muscle, but the osteoma had no contact with the nerve. Following GON decompression, the referred trigeminal and extremity pain completely disappeared. The pain referral from GON entrapment seems to be attributed to the sensitization and hypersensitivity of the trigeminocervical complex (TCC). The clinical manifestations of TCC hypersensitivity induced by chronic entrapment of GONs are diverse when considering the occurrence of extremity pain as well as facial pain.

## 1. Introduction

Frequent pain referrals to the occipital region observed in patients with primary headache are attributed to the convergence of nociceptive afferents from the trigeminal and upper cervical structures to the trigeminocervical complex (TCC) [1]. The nociceptive input from dura mater is transmitted in the ophthalmic division of the trigeminal nerve by the trigeminal ganglion to nociceptive second-order neurons in the superficial and deep layers of the medullary dorsal horn of the TCC [2–4]. The TCC extends from the trigeminal nucleus caudalis to the segments of C2-C3 in the rat [5], cats [6], and monkeys [7]. These dural-sensitive trigeminal neurons show a high degree of convergent input from other afferent sources which typically show facial and corneal receptive fields [1, 8]. Meanwhile, the upper cervical spinal roots also contribute to the sensory innervation of occipital and suboccipital structures [9, 10].

Nociceptive inflow from these structures is mediated by small-diameter afferent fibers in the upper cervical roots terminating the dorsal horn of the cervical spinal cord extending from the C2 segment up to the medullary dorsal horn [9, 10]. The major afferent contribution is mediated by the C2 spinal root that is peripherally represented by the GON [11, 12]. Similar to the trigeminal sensory neurons, these cervical neurons show a high convergence of input from neck muscles and the skin [1, 2]. ,In addition to an anatomical overlap of trigeminal and cervical nociceptive afferents throughout the TCC from the level of the trigeminal nucleus caudalis to at least the C2 segment, direct functional coupling between these afferents was described [3]. These trigeminocervical neurons showed convergent synaptic input from the supratentorial dura mater and from the ipsilateral and contralateral GONs [13, 14].

Occipital neuralgia is defined as paroxysmal stabbing pain arising from the distribution of the greater or lesser occipital nerves and is mostly primary in origin [15, 16]. However, several reports in the last decade suggested that the GON entrapment within the area where the aponeurotic edge of the trapezius muscle attached to the superior nuchal line (the so-called trapezial tunnel) is the most common cause of occipital neuralgia [17–21]. The GON originates in the medial branches of the dorsal rami of the second cervical nerve root and transmits nociceptive afferent information from the temporo-occipital area and vertex, as well as the suboccipital area [17, 18]. Therefore, the GON represents the main sensory afferent traversing through the C2 root, and this afferent information is transmitted directly to the high cervical dorsal horn where the TCC is located [3–5]. It is known that GON entrapment commonly causes aching, tightening, and pressure-like pain in the posterior head and neck, which are GON distributions, as well as occipital neuralgia [22–26]. Also, referred pain of the ipsilateral hemiface was frequently induced for occipital and neck pain due to GON entrapment [22–26]. The chronic, continuous, and noxious afferent inputs of the entrapped GON appear to be associated with sensitization and hypersensitivity of the second-order neurons in the TCC, resulting in referred pain in the trigeminal distribution [22–26].

We report a case of pain involving the ipsilateral extremity along with referred facial pain due to GON entrapment. The ipsilateral extremity pain disappeared along with the associated facial pain following GON decompression.

## 2. Case Report

**Figure 1:**
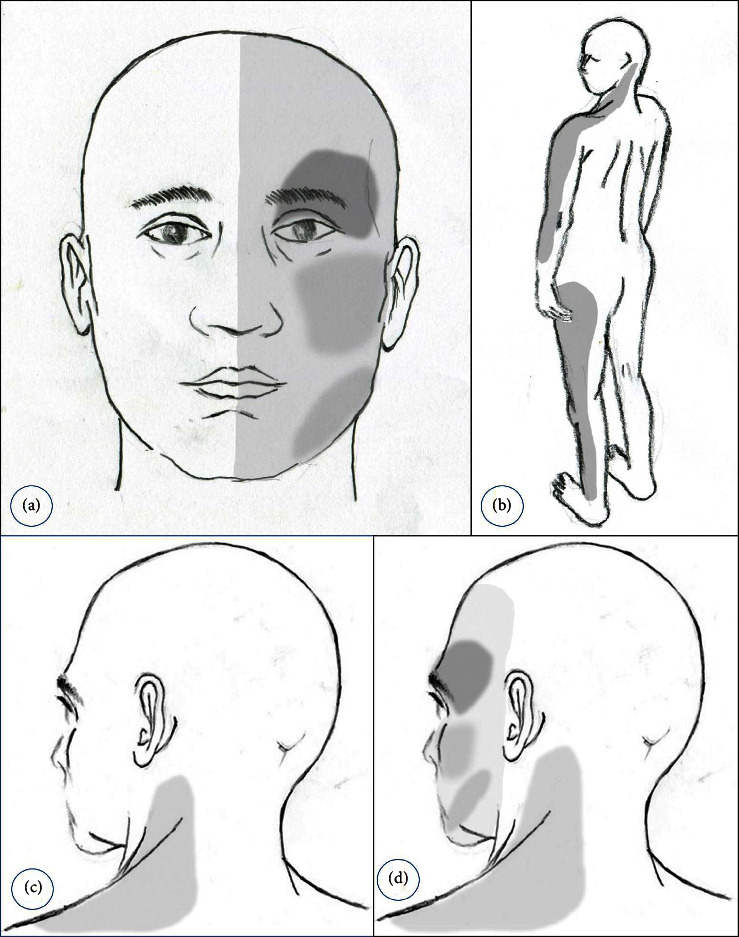
Schematic diagram showing the distribution and characteristics of chronic pain. (a) The gray areas over the left hemiface indicate the distribution of pain that occurred three months prior to the patient's visit. The pain was more severe in the left periorbital, cheek, and chin, and the periorbital area was the most painful. The nature of the pain was a feeling of tightness and pressure, which lasted all day. (b) The location (gray area) of tightening and squeezing pain on the side of the left leg that occurred 5 years ago. A few days after the onset of pain in the left leg, a similar pain occurred in the patient's left arm. (c) The location of the pain in the left posterolateral neck and shoulder that suddenly worsened 3 years before admission. (d) A drawing showing the distribution of pain in the left hemiface (gray area) that suddenly occurred as the existing left posterolateral neck pain worsened. This hemifacial pain was also described by the patient as a constant pulling and tightening pain.

A 49-year-old female patient presented with a three-month history of pain with a sudden onset, persistent tightness, and left-side facial pain, including periorbital, cheek, and jaw areas (Figure 1(a)). The patient's past history was characterized by chronic and persistent pain in the left neck, shoulder, and left leg. The tightening pain was similar to pulling of a rubber band on the left leg laterally and originated 5 years ago without specific antecedent events, including trauma. No pain was detected in the lower back or hip or numbness in the legs. Within a few days after the onset of pain in the left leg, the pain extended to the left arm (Figure 1(b)). This pain persisted throughout the day and was not exacerbated by changes in limb posture, including walking and straining. No abnormalities were found on X-rays and magnetic resonance imaging (MRI) of the lumbar spine and shoulder. The pain in the patient's left arm and leg did not improve despite months of medication and physical therapy. At that time, the pain intensity was evaluated as 2∼4 out of 10 on the numerical rating scale (NRS-11). The pain did not interfere with daily life substantially, and the patient endured it with occasional physical therapies.

About 3 years ago, the patient's left neck and shoulder pain gradually got worse for no apparent reason (Figure 1(c)). The nature of pain was similar to that of the left leg and arm and was expressed as a feeling of persistent pulling and tightness. There was no limitation of motion (LOM) of the neck, and pain did not worsen with neck, arm, and shoulder movements. Repeated MRIs of the shoulder and cervical spine showed no abnormalities. The patient received long-term medications, physical therapy, tender injections, and nerve blocks for more than 6 months, but the pain persisted. The orthopedic surgeons, neurosurgeons, and pain doctors who treated her were unable to arrive at a specific diagnosis. Following months of ineffective medication and physical therapy, she opted for massage therapy for neck, shoulder, and extremity pain. When the pain in the left neck and shoulder was severe, the pain in the leg also worsened. While awake, pain in the left extremity, left neck, and shoulder continued and the intensity of the pain was 3∼5/10, enabling daily life. Because of chronic pain, the patient received medication for 1 year following a provisional diagnosis of myofascial pain syndrome and fibromyalgia by the rheumatologist, without improvement in pain.

Three months before her visit, the pain in the left posterolateral neck suddenly worsened for several days (Figure 1(c)). One day, a tingling sensation and dullness appeared suddenly on her left hemiface (Figure 1(a)). In addition, the patient reports tightness and squeezing pain in the left periorbital and in the left cheek and jaw area. The left periorbital area was particularly painful, requiring pressure with a fist to suppress it (Figure 1(a)). The tightening pain persisted during waking, and its intensity was rated 5∼6 out of 10 on the NRS-11. As the pain in her left hemiface developed, the intensity of pain in the left extremity, neck, and shoulder also increased (Figure 1(d)). Medications such as gabapentin, pregabalin, Ultracet®, and nonsteroidal anti-inflammatory drugs (NSAIDs) including indomethacin prescribed by the patient's rheumatologist were ineffective. She was referred to a neurologist, and neurological examination and MRI of the brain revealed no abnormalities. No organic cause was identified in the referral to ophthalmology and otolaryngology.

The patient's rheumatologist referred her to us for inexplicable left hemifacial and hemibody pain. Despite subjective dullness in her left hemiface, no objective sensory change was confirmed. No allodynia was found. A detailed interview revealed pain in the left ear canal and suboccipital area in addition to pain in the left eye, cheek, and jaw, but there was no pain in the left auricle. No neurological abnormalities were detected including masticatory function in the trigeminal nerve and other cranial nerves. There were no movement restrictions involving the neck, and no physical findings suggestive of cervical radiculopathy were found. There were no abnormal findings on laboratory examination, including the erythrocyte sedimentation rate, C-reactive protein, blood sugar, uric acid, and antinuclear bodies. There was no medical history including hypertension or diabetes other than extremity pain that started 5 years ago.

**Figure 2:**
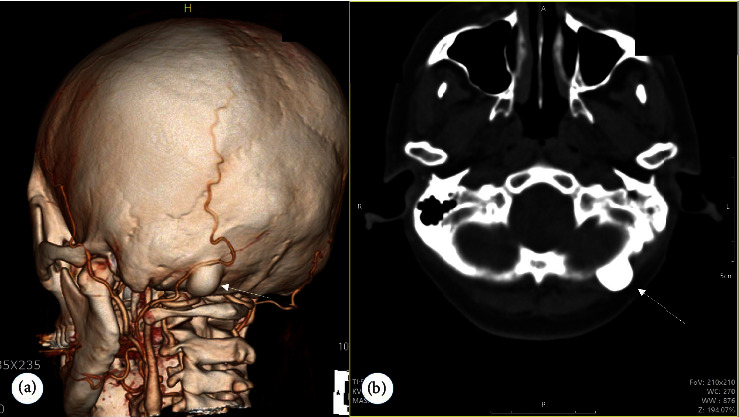
Computed tomographic (CT) scan images showing a single incidental suboccipital osteoma (arrow). A three-dimensional CT scan (a) and an axial CT image (b) of the osteoma. No association was found between osteoma and the greater occipital nerve.

Physical examination revealed a small, nontender, immobile, hard mass, which was palpable in the left suboccipital area (Figure 2). She denied a relationship between the lump and facial pain. In fact, about three years ago, while receiving a neck massage, she heard from the therapist about the existence of a mass. However, she did not request further evaluation because there was no pain, tenderness, or discomfort at the site of the mass. A small calcified mass was observed under the left suboccipital bone during simple skull radiography (Figure 2(a)). Three-dimensional computed tomography and MRI of the brain confirmed a benign osteoma without intracranial extension (Figures 2(b) and 2(c)). Brain MRI showed no structural abnormalities in the cranium including the trigeminal nerve.

With the possibility of referred facial pain due to GON entrapment in mind, occipital nerve block (ONB) of the left GON was performed using 2 mL of 2% lidocaine, which relieved the intensity of the left hemifacial pain by 80% for 2 hours. The same temporary improvement was confirmed at the second ONB, which was performed two weeks later. The second ONB temporarily alleviated the pain in the left neck, shoulder, and left limb for 1 to 2 hours. Three hours after receiving ONB, pain in the left face, neck, and arm continued to persist with the same intensity as before. Due to chronicity and refractoriness of the pain and the possibility of referred facial pain due to chronic entrapment of GON, exploration and decompression of the left GON in addition to excision of the osteoma were recommended after obtaining written informed consent.

**Figure 3:**
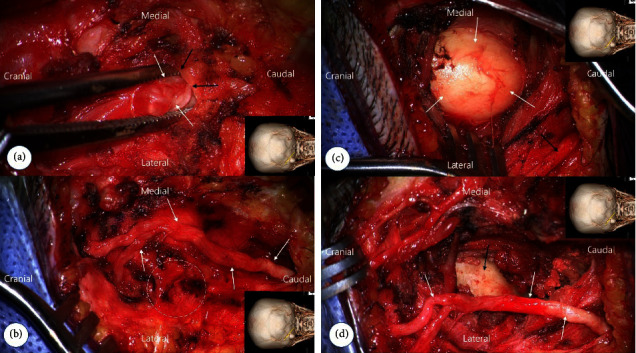
Intraoperative photographs of decompression of the greater occipital nerve (GON) and osteoma removal. (a) GON (white arrows) entrapment at the trapezial tunnel. The GON entrapment is observed by lifting the trapezial aponeurotic edge (black arrows). The inset shows the direction of the image and the location of the incision. The skull reconstruction image in the inset represents the position of the head and skin incision in the surgical field. (b) Complete decompression of the GON (arrows) around the trapezial tunnel. The white circle indicates the location of the osteoma. The GON had no direct contact with the osteoma. (c) Osteoma (white arrow) exposed after retraction of the GON (black arrow). (d) Intraoperative photograph showing the decompressed GON (white arrow) after removal of the osteoma (black arrow). The patient's left hemiface, posterolateral neck, and shoulder pain, as well as pain in the left extremity, had completely improved by 2 weeks after surgery.

In order to expose both the osteoma and the left GON, a 6 cm straight incision was made at an angle in the center of both structures (Figure 3). The incision then involved the trapezius fascia. The GON was found severely entrapped by the fibro-tendinous aponeurotic edge of the trapezius muscle (trapezial tunnel), showing an indentation of chronic entrapment (Figure 3(a)). After the circumferential release of the GON from the tendinous aponeurotic arch, the proximal and distal courses of the left GON were dissected (Figure 3(b)). After retracting the decompressed left GON using a vascular loop, the underlying superior oblique muscle was incised to expose the protruding osteoma (Figure 3(c)). As expected, the osteoma had no direct contact with the GON. The protruding part was removed by drilling (Figure 3(d)).

The effects of GON decompression appeared from the day after surgery. The patient reported that the persistent pain and discomfort in the left eye, cheek, jaw, and half face were reduced by about 70% compared with previous conditions. Simultaneously, it was reported that the tightening pain in the left neck, shoulder, and left arm and leg was no longer felt. Two weeks after the operation, the pain in her left face, neck, shoulder, and limbs disappeared, and the patient did not feel any difficulty in daily life. The patient's occipital discomfort due to the incision was not great either. She refused medications such as gabapentin and Ultracet®. Six months after surgery, the patient reported that she was doing well with no discomfort in her left face and neck. The tightening pain involving her left arm and leg that had lasted for 5 years disappeared.

## 3. Discussion

### 3.1. Convergence and Sensitization in the Trigeminocervical Complex

Nociceptive second-order neurons in the spinal cord can be subjected to transient or long-lasting hyperexcitability in response to afferent stimuli, such as strong noxious stimuli [3, 27]. Increased afferent barrage from primary nociceptive afferents, especially C-fibers, onto second-order neurons is known to be critical in the development of central hypersensitivity in the spinal cord [27–29]. Central hypersensitivity in the nociceptive second-order neurons in the spinal cord was found to be more easily evoked by stimulation of afferents from visceral and deep somatic tissues, such as muscles and joints than cutaneous input [30–32]. The clinical correlates of this central hypersensitivity include the development of spontaneous pain, hyperalgesia, and allodynia [28, 33].

In line with this finding in the spinal cord, central sensitization in the nociceptive neurons in the trigeminal nucleus caudalis, the V homologue of the spinal dorsal horn [34], was demonstrated with the application of a small fiber irritant [35, 36]. Chemical activation and sensitization of meningeal sensory neurons induced a central sensitization of trigeminal second-order neurons in the caudal trigeminal nucleus with a subsequent increased responsiveness to dural and cutaneous facial stimulation [2]. In a population of nociceptive neurons in the laminae V/VI layers of the C2 dorsal horn with convergent input from the supratentorial dura, innervated by the trigeminal nerve, and cervical skin and muscle that are innervated by the GON, stimulation of dural afferent C-fibers increased the background activity, extended the size of cutaneous trigeminal and cervical receptive fields, and decreased the thresholds to mechanical dural stimulation [3, 27]. Furthermore, stimulation of the dura mater led to a sensitization of these convergent neurons with a subsequent increased excitability to neck muscle and GON stimulation [3, 27]. This demonstrates that dural afferents and GON afferents make anatomic connections and these connections are functionally relevant in terms of mutual changes in excitability [1]. Convergence and sensitization in the nociceptive second-order neurons were suggested to be involved in the spread and referral of pain in the trigeminal and cervical area in craniofacial pain and primary headache syndromes [1, 3, 27].

### 3.2. Entrapment of the Greater Occipital Nerve and Pain Referral

The GON originates in the medial branch of the dorsal rami of the second cervical nerve root [17, 18]. It also receives a branch from the dorsal rami of the C3 root [17, 18]. It ascends through the semispinalis capitis muscle and runs rostrolaterally before entering the scalp by piercing the aponeurotic fibrous sling between the trapezius and the sternocleidomastoid muscle near their attachment to the superior nuchal line [17–21]. This aperture (trapezial tunnel) is a frequent site of GON entrapment [19–21]. Compared with other peripheral nerves, the GON widens towards its course to the periphery after piercing the aponeurotic sling between the trapezius and the sternocleidomastoid [18]. This anatomical finding is regarded as relevant to GON entrapment, as widening of the nerve increases the risk of entrapment, especially in the firm trapezius muscle aponeurosis [18].

There is no electrophysiologic evidence from animal experiments on whether entrapment of the GON induces hyperactivity of the nociceptive secondary neurons in the TCC. However, acute stimulation of cervical roots and structures innervated by upper cervical roots in humans, such as the dura mater, vessels, and tumors of the posterior fossa, lead to pain referred to the front of the head [1, 37, 38]. Structures in the back of the head are mainly innervated by the GON that is a branch of the C2 spinal root [39]. The GON constitutes the main sensory afferent nerve through the C2 root, and this afferent input is transmitted directly to the TCC.

“Entrapment neuropathies” refer to isolated peripheral nerve injuries that occur at specific locations where a nerve is mechanically constricted in a fibrous or fibro-osseous tunnel or deformed by a fibrous band [39]. The trapezial tunnel is an anatomical passageway through which the GON must pass and is known as a site where entrapment can occur [17–21]. Entrapment of the peripheral nerve is known to be associated with nerve inflammation and result in pain and headache [40]. Compression injury of the peripheral nerve in a rat model showed inflammation, both local and remote, from the site of peripheral nerve compression [41, 42]. Therefore, it is thought that chronic entrapment of GON induces inflammatory changes, possibly leading to sensitization and hypersensitivity of secondary neurons in the TCC. Although chronic GON entrapment itself is an individual's constitutional problem, the degree of compression seems to vary from person to person [25].

Indeed, referred trigeminal pain caused by GON entrapment in the trapezial tunnel has been reported in several recent reports. Pain referral to the facial trigeminal distribution triggered by GON entrapment occurred not only in the *V*1 region but also in the *V*2 and *V*3 regions and even caused hemifacial pain and sensory changes [22–26]. We frequently found immediate disappearance of the referred trigeminal facial pain after GON decompression [22–24]. These reports are considered direct clinical evidence that the chronic, continuous, noxious afferent input of GON entrapment may be associated with sensitization and hypersensitivity of the second-order neurons in the TCC.

The patient's left hemifacial pain was presumed to be a sudden onset of referred facial trigeminal pain due to chronic GON entrapment. The authors have already experienced many cases of referred trigeminal pain manifesting suddenly in the facial half in many patients with GON entrapment [14–18]. In addition, pain within the ipsilateral ear canal and lateral neck was frequently reported by patients with GON entrapment [15, 16]. In addition, pain in the suboccipital area was noted along with posterolateral neck pain. By the ONBs, which was performed with the possibility of GON entrapment in mind, not only neck pain but also ipsilateral facial and extremity pain were improved temporarily.

### 3.3. Referred Pain to the Extratrigeminal Area

The current case is unique in that the referred pain due to GON entrapment extended not only to the face, where it occurs frequently, but also to the extremities. The nondermatomal pain in the limbs was not accompanied by objective neurological or musculoskeletal abnormalities. The pain in the left arm and leg disappeared promptly along with the referred pain involving the left hemiface with GON decompression. Considering the pathogenesis of referred trigeminal facial pain due to chronic GON entrapment based on the convergence of trigeminocervical afferents and subsequent sensitization of secondary neurons in TCC [1, 3, 27], the neuroanatomical basis of pain referral to the extremities is attributed to GON distribution and connectivity. The fact that hemifacial pain, which occurred additionally in chronic neck, shoulder, and limb pain, disappeared due to GON decompression means that GON entrapment caused hypersensitivity of the TCC.

It has been shown that caudal trigeminal nucleus (Sp5C) trigeminovascular neurons receiving convergent input from the dura and periorbital area not only increase their responses following the application of inflammatory agents to the dura but also become sensitized for several hours [43, 44]. Sp5C-sensitized neurons have lower thresholds to both dural and periocular skin stimulation and show a significant increase in the size of their dural and cutaneous receptive fields [2]. Based on these findings, it was proposed that the referred, cutaneous allodynia observed in migraine patients is due to central sensitization of Sp5C neurons following peripheral sensitization of meningeal nociceptors [2]. Moreover, the ophthalmic region of Sp5C, which contains neurons that receive convergent input from the dura and periorbital skin, sends projections to the ophthalmic primary afferent projection area of the contralateral trigeminal brain stem sensory complex [44]. These projections are somatotopically organized and extend rostrocaudally from the caudal spinal trigeminal nucleus to the upper cervical dorsal horn C2–3 segments [44, 45]. Contralateral projections could provide input that specifically modulates the activity of medullary and spinal dorsal horn cells driven from the ophthalmic division of the trigeminal nerve [43]. Such input could become effective following long-lasting noxious stimulation of meningeal nociceptors and thus contribute to the central sensitization that occurs after long-lasting migraine attacks [46]. Sp5C also projects to the ipsilateral junction of Sp5C and interpolaris (Sp5I), oralis (Sp5O), and principal sensory (Pr5) nuclei over their whole caudal-rostral extent [47]. Ipsilateral input from Sp5C neurons to rostral trigeminal nuclei could contribute to the amplification of nociceptive output to supramedullary structures via the interpolar, oral, and principal subdivisions since these regions convey orofacial input to the brain stem and thalamic areas [48].

Following trigeminal nerve injury (or inflammation), the changes in the excitability of afferents may not be limited to the damaged afferents [49]. The excitability of undamaged afferents may also be altered. Changes may also occur in the peripheral innervation patterns of afferents spared from the nerve injury. As a result, an increased pain sensitivity reflected in extraterritorial secondary hyperalgesia may occur [49]. The interactions between satellite glia cells and neurons involve the release of chemical mediators and changes in signaling mechanisms that can result in the spread of hyperexcitability throughout the different trigeminal divisions of the trigeminal ganglion [49, 50]. For example, injury to primary afferents in the infraorbital nerve, a branch of the maxillary division of the trigeminal nerve, may increase the excitability of not only primary afferent neurons in the maxillary division but also neurons in the other two trigeminal divisions of the ganglion [49]. Meanwhile, transection of the upper cervical spinal nerves in rats resulted in mechanical allodynia and thermal hyperalgesia in the facial skin, phosphorylated extracellular signal-regulated kinase (pERK) expression, and astroglial cell activation in the rats [51]. This suggested that extraterritorial facial pain can occur in the upper cervical nerve injury [51]. Therefore, the convergence of cervical and trigeminal afferents onto caudal trigeminal nucleus and upper cervical dorsal horns is thought to be a mechanism in orofacial pain related to neck injury. Chronic entrapment of the GON does not cause the same nerve damage such as nerve transection, but it is thought to have the potential to induce nerve inflammation, leading to hypersensitivity of the TCC.

Nociceptive transmission from the central endings of the nociceptive primary afferents to neurons in trigeminal brainstem sensory nuclear complex (TBSNC) may take place in the rostral components of the TBSNC (e.g., Sp5O) but is especially evident in its more caudal components (Sp5C), the medullary dorsal horn, also known as caudal trigeminal nucleus and caudalis-interpolaris transition zone, and in the upper cervical dorsal horns [47, 49]. The nociceptive transmission process involves the release of several neurochemicals, such as glutamatergic, neurokinin (e.g., substance P and CGRP), and purinergic mediators, from the central endings of the primary afferents. In the situation where there are abnormal hyperexcitable or ectopic afferent inputs, neuroplastic and glioplastic changes may be produced in the TBSNC [49].

Central sensitization of the TBSNC, along with peripheral sensitization reflecting hyperexcitability of afferent inputs, is thought to be consistent with the sensory experiences of human subjects under acute or chronic experimental orofacial pain conditions [49, 52]. A typical example is temporomandibular disorders (TMDs). They typically displayed allodynia an hyperalgesia and complicated by one or more comorbidities such as depression, anxiety, stress, sleep disturbance, and comorbid pain, as well as extraterritorial sensory abnormalities and spread of sensitivity (ETSS) to widespread areas innervated by the trigeminal nerve or even tissues supplied by upper cervical nerves [49, 52]. Unilateral transection of the medial branch of the infraorbital nerve supplying the medial upper lip and anterior teeth induced hypersensitivity in not only ipsilateral face but also ETSS lasting several weeks in extraterritorial trigeminal areas (e.g., contralateral ear and upper lip and ipsilateral lower lip) and even in nontrigeminal regions such as the hindpaw [49, 52, 53]. Therefore, ETSS can occur in different body regions in some chronic orofacial pain conditions. These clinical features are thought to be related to neuroplasticity and glioplasticity expressed as central sensitization occurring in the TBSNC and other brainstem areas (e.g., rostral ventromedial medulla and reticular formation) that receive trigeminal nociceptive inputs and send modulatory influences to areas of the spinal cord (e.g., spinal dorsal horn) [49]. Sensitized neurons of TBSNC may influence spinal nociceptive circuits and related behavior [49, 54].

The rostral ventromedial medulla (RVM) plays a major role in the endogenous modulation of nociceptive transmission in the spinal and trigeminal nociceptive pathways in the central nervous system [55]. It receives spinal and trigeminal somatosensory afferent inputs that include nociceptive inputs relayed through the spinal dorsal horn, medullary dorsal horn (also known as Sp5C), parabrachial nucleus, locus coeruleus, and subnucleus reticularis dorsalis and receives descending projections from higher brain centers such as midbrain periaqueductal gray (PAG) and hypothalamus [54, 55]. The major projection sites of the RVM include spinal and medullary dorsal horns, and stimulation of the RVM modulates nociceptive behavior and the activity of the spinal dorsal horn and nociceptive neurons in the medullary dorsal horn [54, 55]. Noxious thermal stimulation of the tail or perioral skin in the rat evoked the tail-flick response (TF) and jaw-motor response (JMR), and the latency of the JMR was significantly shorter than that of the TF. Heating at either tail or perioral skin caused an increase in ON-cell activity and a decrease in OFF-cell activity before the occurrence of the TF and JMR [54]. The activity of RVM is closely related to the activity of the medullary dorsal horn and plays an important role in the elaboration of diverse nociceptive behaviors evoked by noxious stimulation of widely separated regions of the body [56]. In connection with the central sensitization of Sp5C by trigeminal and high cervical excitatory inputs, it is likely that systemic pain may be caused by changes in RVM activity, which is closely related to the nociception processing of Sp5C.

Meanwhile, headache-related extracephalic pain referral to the upper and lower extremities has already been observed for a long time in migraine, a primary headache [56]. In fact, central sensitization and hyperactivity caused by noxious peripheral input have been demonstrated to occur not only in secondary neurons in the TCC but also in the third-order thalamic neurons that receive convergent information from the head and forearms [57, 58]. It has been confirmed that cutaneous allodynia during migraine attacks occurs not only in the referred pain area of the ipsilateral head but also in the nonreferred pain area in the ipsilateral head and extracephalic arm during migraine attacks [58]. Hypersensitivity of the third-order thalamic neurons can occur with stimulation of the large skin areas on both sides of the body following a small unilateral injury [59]. Thus, in migraine headaches, the importance of peripheral sensitization, as well as central sensitization, is emphasized [60, 61]. The extremity pain observed in the present case can also be interpreted as the occurrence of hypersensitivity of the thalamic third neurons, which is thought to be the pathophysiology of extension of cutaneous allodynia observed in migraine patients.

## 4. Conclusions

We report a case of referred extremity pain due to chronic GON entrapment that preceded referred trigeminal facial pain. The unexplained pain in the extremities lasted for 5 years without any specific cause identified via multiple tests. It was impossible to suspect an association with GON until later neck and facial pain appeared. This case presents diverse clinical manifestations of referred pain triggered by the sensitization of the TCC following chronic entrapment of the GON. GON entrapment may induce sensitization of TCC, which may affect brainstem and thalamus activity involved in nociceptive information processing in the central nervous system, resulting in associated pain in the extremities and ipsilateral face.

## Data Availability

No data were used to support the study.
